# Adaptation, further development and evaluation of the measurement properties of the person-centred community care inventory (PERCCI-S) for use in the Swedish municipal health care system

**DOI:** 10.1186/s12913-025-13178-7

**Published:** 2025-07-30

**Authors:** Theresa Larsen, Richard Sawatzky, Helle Wijk, Ewa Wikström, Axel Wolf

**Affiliations:** 1 The Gothenburg Region, Gothenburg, Sweden; 2 Institute of Health and Care Sciences at the University of Gothenburg, Gothenburg, Sweden; 3 Centre for Person-Centred Care, Sahlgrenska Academy at the University of Gothenburg, Gothenburg, Sweden; 4 School of Nursing, Trinity Western University, Langley, Canada; 5 Centre for Advancing Health Outcomes, Providence Health Care Research Institute, BC Vancouver, Canada; 6 Faculty of Nursing, University of Alberta, AB Edmonton, Canada; 7 Department of Architecture and Civil Engineering, Chalmers University of Technology, Gothenburg, Sweden; 8 Department of Quality Assurance, Sahlgrenska University Hospital, Gothenburg, Sweden; 9 Department of Management & Organization, The School of Business, Economics and Law, University of Gothenburg, Centre for Health Governance, Gothenburg, Sweden

**Keywords:** Home-based primary care, Swedish municipal home-based health care, Person-centred care, Measurement evaluation, Patient reported experience measures (PREM), Psychometrics, Mixed-methods approach

## Abstract

**Background:**

Successful implementation and sustainability of person-centred care (PCC) require continuous evaluations and valid measurements. While several instruments measure patients’ experience with PCC, to our knowledge, no validated instrument exists in Swedish for use in home-based primary care (HBPC). This study aimed to adapt and further develop an instrument for measuring patients’ experiences of person-centred care in HBPC for use in the Swedish municipal health care system, with a 12-item version of the Person-Centred Community Care Inventory (PERCCI) used as a starting point. Furthermore, its content and measurement properties were evaluated via a mixed-methods approach involving item response theory and qualitative content analyses.

**Methods:**

This study was conducted in two stages. First, the PERCCI 12 item version was translated into Swedish using a forward-backward approach. Content validity was evaluated through focus groups with 24 registered nurses and managers, resulting in revisions. Second, the revised version (PERCCI-S) was psychometrically evaluated via two rounds of postal questionnaires (2022; 2023) with patients 18 years or older receiving municipal HBPC in Sweden (*n* = 1,171; *n* = 1,429). The psychometric evaluation involved factor analyses and item response theory analyses to assess dimensionality, item difficulty and discrimination, item and test information, test‒retest reliability, internal consistency reliability, as well correlational analyses of convergent and discriminant validity. Content validity was further assessed through a panel review with experts (*n* = 7) and cognitive interviews with patients (*n* = 20).

**Results:**

Exploratory and confirmative factor analyses support an overall unidimensional structure. The item response theory analyses indicate acceptable item characteristic curves and overall test information. The internal consistency reliability was satisfactory (*r*_2022_ = 0.97 and *r*_2023_ = 0.96). Test-retest reliability showed good temporal stability (*r* = 0.79, *n* = 96). The content validity index was 1.0, indicating that all the items were relevant. However, the scale’s discriminant validity was unsatisfactory, with 18.0% of respondents having the highest score.

**Conclusions:**

The psychometric evidence of the PERCCI-S provides support for its use in the Swedish municipal HBPC. Future studies should test different response formats in an effort to reduce ceiling effects.

**Supplementary Information:**

The online version contains supplementary material available at 10.1186/s12913-025-13178-7.

## Introduction

Health care systems around the world are amidst a major transition towards integrated care solutions where more care is provided in open forms and in the patient’s home instead of in hospitals and care institutions [1–4]. The transition emphasizes that health and social care systems should be person-centred on the basis of patients’ needs instead of organizations’ needs and conditions [5–7]. According to Swedish law [8], the design and execution of health and social care interventions should be person-centred in terms of being determined in partnership with the person in need of care as much as possible. The objective is to involve patients in cocreating their care and treatment procedures, thereby fostering shared responsibility, empowerment, and considering the patient as an active participant and partner in their own care [9, 10]. 

The successful implementation and sustainability of person-centred care (PCC) requires continuous evaluations and valid measurements for determining the extent to which the goals of PCC are being achieved [11]. Despite its central role, evaluations of PCC remain limited, particularly in home-care services, which differ significantly from traditional institutional care. Several generic patient-reported experience measures (PREMs) as well as instruments that specifically measure patients’ experiences with PCC have been developed over the years. In general, while both focus on patients’ experiences, generic PREMs capture patients’ subjective evaluations of their care experiences in general, whereas PCC instruments aim to assess the alignment of care delivery with the principles of person-centredness, for example, patient-caregiver partnership and empowerment [12]. However, there is a lot of overlap given that most PREMs also measure aspects central to person-centredness, such as respect, coordination and emotional support.

Ten years ago, de Silva [13] identified more than 200 validated instruments in a review of the evidence about commonly used approaches and tools to help measure PCC, and since then, many more instruments have been developed and evaluated. Instruments that measure PCC have almost exclusively been used in residential and institutional settings despite the shift in the global paradigm to integrated person-centred home-based health care [1, 2]. Systematic reviews highlight the lack of measures suitable for home-based primary care (HBPC), suggesting that these tools are important research areas [14, 15]. 

In Sweden, primary care is delivered by both regions and municipalities. Municipal health care in Sweden refers to care and treatment that, through the municipality’s commitment and responsibility, is given in nursing homes, day care and home health care in ordinary housing “but not such health care given by a doctor” [16]. The largest professional groups working in Swedish municipal health care include registered nurses, occupational therapists, and physiotherapists. Additionally, dieticians, pharmacists, and assistant nurses sometimes also work in municipal health care. Most interventions, however, are delegated to assistant nurses, care assistants, and medically untrained staff employed in social care and nursing homes (i.e., not the same organisations as HBPC [17]. Common interventions in municipal health care include wound dressing, medication injections, catheter care and fitting of assistive devices such as a wheelchair and a walker, but more advanced interventions such as peritoneal dialysis, blood transfusions, and intravenous antibiotic treatment are also administred [18]. In 2023, almost 414 000 people received municipal health care in Sweden, the majority being 80 years or older [19]. The number increases every year due to the growing number of elderly citizens, medical and technological advancements that enable more home-care, and political policies emphasizing that primary care should be the hub of health care [18]. 

Although close to half a million patients receive municipal health care in Sweden annually, there is a lack of relevant and quality-assured data that can be used to evaluate municipalities’ performance in a satisfactory manner [20, 21]. For example, there is no nationwide follow-up of patients’ experience with municipal HBPC and no validated instruments in Swedish that can be used to measure PCC from the perspectives of patients in home-care services [22, 23]. This lack of data creates challenges for policymakers, health care managers, and professionals in identifying areas for improvement and ensuring high-quality, person-centred care.

The limited understanding of the quality of person-centred care from the patient’s perspective prompted municipal health care and social care managers in the Gothenburg region in western Sweden to commission patient surveys in 2022–2023 to measure PCC in HBPC. The first author was the project leader of these surveys.

A literature review was conducted to find an existing measure that could be used in the surveys [24]. Generic PREMs were found to insufficiently address key components of PCC or the long-term care relationship between care workers and patients, which are central to HBPC, as they typically focus only on the most recent care experience. Similarly, generic PCC instruments often fail to capture the unique dynamics of providing care in patients’ homes or the continuity of the care relationship, instead tending to concentrate on isolated, recent care encounters. Existing measures were also found to not work well in the context om Swedish municipal HBPC where the care is provided by several different professions employed in different organizations. This fragmentation underscores the need for an instrument specifically tailored to the Swedish municipal HBPC setting.

Two validated instruments in English were identified that focus specifically on PCC in home-care services: the Client Centred Care Questionnaire (CCCQ) [25] and the Person-Centred Community Care Inventory (PERCCI) [26]. The two instruments were discussed with a reference group consisting of municipal health care and social care representatives. The group concluded that the two instruments are quite similar but that the PERCCI has a stronger focus on home-based *health* care, whereas the CCCQ focuses more on *social* care, thus making the PERCCI the most suitable choice for use in Swedish municipal HBPC.

The PERCCI is a questionnaire originally designed through a participatory process in which older people living in the community and receiving a blend of mental health and social care services in Great Britain were asked to describe good and bad care experiences. The 131 statements resulting from this process were mapped to three overarching themes conceptualizing person-centeredness: (i) understanding the person; (ii) engagement in decision-making; and (iii) promoting care relationships, identified in a literature review-based concept synthesis [27]. The final questionnaire, consisting of 18 items, had excellent validity and reliability properties on the basis of a sample of nearly 600 older people [26]. In 2020, Wilberforce subsequently published a shorter, 12-item version of the PERCCI online [28]which has not yet been validated.

The 12-item version of the PERCCI was chosen for this study over the validated 18-item version because shorter questionnaires are easier to use, especially for patients receiving municipal HBPC in Sweden, where the majority are over 80 years old and have significant functional impairments and multimorbidity. Also, the 18-item version, but not the 12-item version, included three negatively phrased items, which is generally not recommended in an otherwise positively stated questionnaire, as it leads to ambiguity and less reliable results [29]. 

The aim of this study was to adapt and further develop an instrument for measuring patients’ experiences of person-centred care in HBPC for use in the Swedish municipal health care system, with the 12-item version of the PERCCI used as a starting point. Furthermore, its content and measurement properties were evaluated via a mixed-methods approach involving both psychometric and qualitative content analyses.

## Methods

### Study design

The study was conducted in two phases. In the first phase, the PERCCI 12-item version was translated into Swedish with the permission of the instrument developers. Content validity was evaluated through focus groups with registered nurses and managers working in municipal health care in Sweden, resulting in some revisions of the instrument [30] (Additional File 1).

In 2022, the first author was commissioned to include measures of PCC from the patient’s perspective in the annual Gothenburg Region’s key figure report on integrated municipal health care [23]. The revised Swedish version of the 12-item PERCCI (called the PERCCI-S) was used for this purpose. Up until this time, the purpose of the work on the Swedish version of PERCCI had not been to validate the instrument, but to measure PCC to improve care quality.

**Fig. 1:**
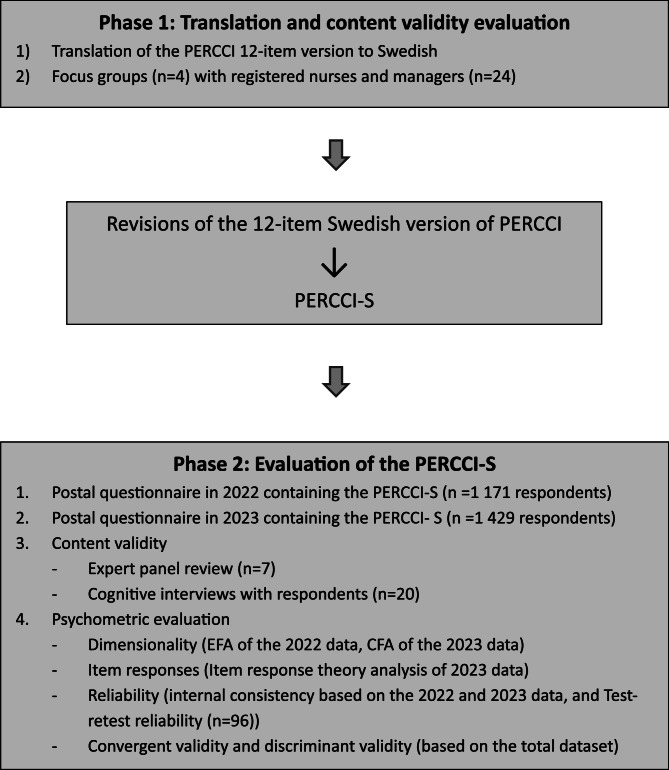
Design of the study

In the second phase, the PERCCI-S was evaluated via two rounds of postal questionnaires with patients 18 years or older receiving municipal HBPC in western Sweden. Content validity was evaluated through a panel review with experts and cognitive interviews with patients. The psychometric evaluation involved (a) exploratory and confirmatory factor analyses to assess dimensionality; (b) item-response theory analysis to assess item characteristic curves and statistical information; (c) internal consistency (ordinal alpha) and test-retest reliability analyses; and (d) convergent and discriminant validity analyses. The study design is presented in Fig. 1.

### Translation

The PERCCI 12-item version was translated independently into Swedish in a cyclic process [31] of translation and back-translation by two bilingual persons on the research team, with English as their second language and Swedish as their mother tongue. One of the researchers was well acquainted with PCC and the other in Swedish municipal HBCP. An evaluation of conceptual equivalence, cultural relevance and comprehensiveness between the original English version and the translated Swedish version was conducted by a third bilingual person, with Swedish being the person’s second language and English being the person’s mother tongue, resulting in some minor changes in the wording of some items. In contrast to the first two translators, the third person lacked prior experience with HBPC and PCC instruments. The translation resulted in a preliminary Swedish PERCCI 12-item version (Additional file 1).

### Focus groups with professionals

The Swedish PERCCI 12-item version was discussed in four focus groups with a total of 24 nurses and managers working in four different municipalities in western Sweden. In the focus groups, the participants reflected on the relevance and understandability of each PERCCI item and whether PERCCI could be a useful tool for measuring patients’ experiences with HBPC provided in the Swedish municipal healthcare system.

Written notes were taken during the focus groups by a research assistant as well as the first author of this paper, who also led the discussions in the focus groups. The data were analysed via qualitative content analysis [32]. 

### Surveys in 2022 and 2023: setting and sampling

A postal questionnaire including the PERCCI-S was distributed in two rounds to patients 18 years or older receiving HBPC. In 2022, the questionnaire was distributed to all patients 18 years or older receiving HBPC in September 2022 in 8 municipalities in western Sweden (*n* = 1 594). We also randomly selected 975 patients 18 years or older receiving HBPC, regardless of illness or disability, in September 2022 in another 3 municipalities in western Sweden (total *n* = 2 569). In 2023, the questionnaire was distributed to all patients 18 years or older receiving HBPC in October 2023 in 8 municipalities in western Sweden (*n* = 1 539). In October 2023, we randomly selected another 1 568 patients 18 years or older receiving HBPC care, regardless of illness or disability, in 4 other municipalities in western Sweden (total *n* = 3 107). Some of the patients who replied to the questionnaire in 2023 were the same as those in 2022. The exact number is unknown because this information was only available for seven of the ten municipalities that participated in both 2022 and 2023, which identified 236 patients who had replied to the questionnaire in both years (i.e., 25% of the patients in these municipalities who replied to the questionnaire in 2023 also replied in 2022).

The questionnaires in both 2022 and 2023 contained the PERCCI-S as well as a question on how satisfied the respondents felt overall with the municipal HBPC. The questionnaire also included background questions about age, gender, general state of health, problems with worry or anxiety, cohabitation, whether the patient also had social care services (for example, help with cleaning, cooking or personal hygiene), how often the patient received care from the municipality and whether someone other than the patient assisted with answering the questionnaire. The questionnaire in 2023 also included a question on how long the patient had received municipal HBPC.

The questionnaire and written information about the study was distributed via postal mailing. The patient could choose to answer a digital version of the questionnaire or a paper version and post it in a prepaid envelope addressed to the first author of this article. Every patient was given an identification code, and the code was printed on the questionnaire. This made it possible to identify which patients had replied to the questionnaire. Only a contact person in each municipality had access to the code key. The contact persons in the municipalities were required to sign a protocol certifying that they had followed the procedure for conducting the patient survey as outlined by the researchers in the project.

### Content validity

#### Cognitive interviews with patients

Content validity was assessed through cognitive interviews [33–35] with 19 patients and one relative via telephone a few months after they replied to the questionnaire. In the 2022 questionnaire, we asked respondents if they would like to be interviewed about the questionnaire and municipal HBPC. If they were, they were asked to send their telephone number to the first author of this paper. Fifty people volunteered. The first 20 on the list who replied when calling were interviewed.

The interviewer was unknown to the patients prior to the interviews and was not involved in any aspect of the patients’ care. The interviewer read the items of the PERCCI-S questionnaire, and the patients were asked to “think aloud” when replying and to verbalize their thought process (e.g., “Tell me what you are thinking about…” or “How are you coming up with your answer to the question?”). To confirm that patients’ interpretation of each item was consistent with the intended meaning, patients were asked to describe their understanding of the instructions and listed concepts and to confirm the clarity and relevance of the items provided in the questionnaire. Patients were also asked to confirm their understanding of the response options for the other questions in the questionnaire and to confirm whether any items or response options should be added or removed.

The interviewer used a protocol based on the questionnaire to note comments from each patient while he or she responded to the questionnaire. All the interviews were audio-recorded and transcribed intelligent verbatim [36]. The transcripts were analysed using qualitative content analysis [32]. During cognitive debriefing, patient feedback was coded and analysed to identify any issues related to interpretation, clarity, relevance, acceptability, appropriateness, conceptual overlap, time frame, response options, missing concepts and meaningful change.

#### Expert panel

Content validity was also investigated through an expert panel and by computing a content validity index (CVI) [37, 38]. The expert panel consisted of four researchers with expertise in PCC and three practitioners working in municipal health care as operations developers, managers, or chief nursing officers. All the experts were registered nurses and had long-term experience working in municipal health care or hospital care. The experts were asked to individually rate the relevance of each item on a four-point scale: 1 = “Not relevant,” 2 = “Somewhat relevant,” 3 = “Quite relevant,” and 4 = “Highly relevant.” They were also encouraged to write comments on individual items and the overall instrument. The results were discussed with the expert panel. Discussions were led by the first author, who also took notes during the meeting. The item CVI (I-CVI) was calculated by dividing the number of experts who rated each specific item as quite relevant or highly relevant by the total number of items. An I-CVI value above 0.8 indicates satisfactory content validity [39]. 

### Psychometric evaluation

Patient characteristics, item and total-score distributions, and missing data patterns for the PERCCI-S were assessed using descriptive statistics. Total-score distributions were evaluated for ceiling effects, defined as occurring when over 15% of respondents attain the lowest or highest possible scores [40]. The items were treated as ordinal for all analyses.

The dataset from 2022 was analysed using exploratory factor analysis (EFA) to determine the number of underlying factors in the PERCCI-S. Factors were extracted using the weighted least squares mean and variance adjusted (WLSMV) estimator, and oblique (Geomin) rotation was used to assess the pattern of factor loadings. The number of factors was determined on the basis of a combination of indicators, including statistical information (eigen values, factor loadings, and model fit) and clinical knowledge. Model fit was examined on the basis of (a) the root mean square error of approximation (RMSEA), with values of < 0.06 or 0.08 being indicative of good or acceptable fit, respectively; (b) the comparative fit index (CFI), with values < 0.95 being indicative of good fit; and (c) the pattern of residual correlations [41]. Larger sample sizes, as in this study, can lead to inflated chi-square values, making it more likely to reject the model even if the model provides a reasonable fit to the data. Therefore, relying solely on chi-square values to evaluate model fit in large samples may not be sufficient, and additional fit indices that are less sensitive to sample size, such as the CFI, Tucker‒Lewis index (TLI) and RMSEA, are often used in conjunction to provide a more comprehensive assessment of model fit [41]. 

The PERCCI-S was subsequently evaluated via confirmatory factor analysis (CFA) of the 2023 data to confirm the dimensional structure and internal consistency reliability identified through the EFA performed on the 2022 data. The same estimation procedures and fit criteria were applied as described above. Model fit was also evaluated in terms of the strength of factor loadings and patterns of residual correlations [42]. Additionally, item response theory analyses were conducted to further assess the psychometric characteristics of the items. Specifically, Samejima’s 2-parameter graded response model was fit to the data to assess item discrimination and difficulty, item characteristics and information curves, and overall test information [43, 44]. 

Internal consistency was assessed with ordinal alpha [45]. The measure can be interpreted like the traditional Cronbach’s alpha; that is, values > 0.7 are commonly recommended in the literature [45]. Additionally, test-retest reliability was assessed for 96 respondents completing all items at both T1 and T2 with intraclass correlation (ICC, two-way mixed-effects model, absolute agreement, single measure) [46]. The mean time elapsed between T1 and T2 was approximately 3 weeks. There are no standard values for acceptable reliability using ICC, but the conventional criterion is 0.7 or more [40]. 

Convergent and divergent validity were assessed by examining the correlations of the total PERCCI-S score with “How satisfied are you overall with municipal home-based health care?”, perceived overall health, problems with worry or anxiety and the frequency of home care. For this purpose, Kendall’s tau-b correlation analysis was used. On the basis of previous research on the impact of patient characteristics and PREMs, we expected the correlation of the PERCCI-S with overall satisfaction with municipal home-based health care to be relatively greater (indicating convergent validity) and relatively lower for overall health, a higher frequency of worry or anxiety and a higher frequency of home care (indicating divergent validity) [47]. 

Factor analyses were conducted using pairwise deletion, and item response theory analyses were conducted using full information maximum likelihood, following recommendations by Enders [48]. The descriptive statistics (e.g., respondent characteristics and distribution of item categories) were calculated in IBM SPSS, version 29.0.0.1. Most latent variable analyses and multiple imputations were carried out in Mplus software, version 8.10 [49]. COSMIN guidelines were followed for interpreting and reporting the results [40, 50, 51]. 

### Methods for handling missing data

Users of the PERCCI-S in municipalities usually do not have the statistical knowledge or tools to perform multiple imputations when handling missing data; thus, for the total PERCCI-S score to be usable in municipalities, and not only in research, an alternative model for handling missing data must be found. To find the most appropriate way to handle missing data, a comparison of the mean PERCCI-S scores was performed via different methods: (a) listwise deletion (assuming data were missing completely at random), (b) missing data replaced by the average mean for those respondents scoring at least half of the items, and (c) item-level multiple imputation of 10 data files (assuming data to not be missing at random).

### Ethical considerations

This study was conducted according to the principles of the 1996 Declaration of Helsinki [52]. Ethics approval for the study was obtained from the Swedish Ethical Review Authority (Dnr 2020 − 01869, 2022-03400-1, 2022-05722-02, 2023-04968-01 and 2023-04968-02). All participants were informed about the study and gave consent to participate. All participants received written information about the study. The participants in the cognitive interviews and focus groups also received verbal information. Participation was voluntary, and participants could withdraw from the study at any time. In the questionnaires, the participants consented by submitting the questionnaire.

## Results

### Development of PERCCI-S

Based on the results from the focus groups with professionals, changes in the formulation of four items in the first Swedish version of PERCCI were made to avoid misunderstanding and to better match the Swedish context of municipal HBPC.

Item 4 (I have developed a close connection with them) was changed to “I have confidence in the care workers”. Participants in the focus groups emphasized the importance of maintaining a professional relationship between health care workers and patients, rather than fostering overly personal connections. Item 6 (I’m given enough time to say what I want to say) was changed to “I’m given enough time to say what I want to say about my health and my care”. The participants highlighted the need to specify the context in which patients feel they are being given sufficient time to express themselves, ensuring clarity and focus on health and care-related discussions. Item 9 (I am helped to stay in touch with my local community) was changed to “The care workers help me coordinate my care”. In Sweden, supporting patients in staying connected with their local community falls under the responsibility of social care services, not municipal health care. Coordination of care, however, is a key component of PCC [9, 10] and Sweden’s transition toward more integrated care [4]. To address this gap, participants suggested the rephrased wording, as care coordination was missing from the original PERCCI framework. Item 12 (My care and support helps me to build confidence) was modified to “My care and support strengthens my ability to manage my illness and treatment”. When discussing this item, participants stressed the need to specify the area in which confidence is being built. They argued that the goal should not be to instil general confidence but to enhance patients’ ability to manage their health conditions and treatments effectively.

The revised version (PERCCI-S) consists of 12 items rated on 4-grade Likert scale with the response options “never or rarely”, ” sometimes”, “often” and “always”. Total score ranged from 12 to 48 (Additional file 1).

### Content validity

#### Expert panel

Results from the qualitative content analysis based on experts show that all participants found the PERCCI-S to cover the most important aspects of PCC in municipal HBPC. Participants rated all the PERCCI-S items as “Quite relevant” or “Highly relevant”, resulting in an I-CVI value of 1.0. Items 1, 6, 11 and 12 were rated as “highly relevant” by all the experts, and Items 4, 8 and 10 were rated as “highly relevant” by all but one expert, who rated them as “quite relevant”. Item 9 was rated as “highly relevant” by four experts and “quite relevant” by three experts. Items 2, 3 and 5 were rated as “highly relevant” by three experts and as “quite relevant” by four experts.

Although the CVI analysis indicated excellent consensus among the experts, the participants still proposed revisions to improve the clarity of some items. To better align with the contemporary Swedish concept of person-centred care (PCC) [10, 53]which emphasizes the patient as an active partner in the care process, some experts suggested rewording items 8 and 11 to portray the patient as an equal partner with healthcare professionals in achieving care outcomes. Additionally, one expert recommended including instructions for respondents to base their answers on a specific time period, arguing that this addition would enable municipalities to interpret the results more effectively and use them as a foundation for decision-making.

#### Cognitive interviews with patients

The interviewed respondents were 48–93 years old. Nine were women, and 11 were men. They lived in 9 different municipalities in western Sweden. Most of the respondents scored high on the PERCCI (i.e., they perceived the care as person-centred and were overall very satisfied with the care), but a few of the respondents scored low.

Results from the interviews show that most of the respondents could differentiate between municipal HBPC and social care and which staff performed what services. The interviews also revealed that most of the respondents felt that the PERRCI-S covered the most important aspects concerning the quality of municipal HBPC. However, some respondents mentioned that they feel that too many different persons perform care in their home and that the measurement would benefit from adding an item on care continuity. Others described that health care workers are not always informed about their situation and care needs: “Some do not have a clue what to do, and I have to show them and explain everything”.

A few of the respondents explained that they had some difficulties in replying to the questionnaire because the quality of care varies greatly between different health care workers: “they are all very kind, but some are not from Sweden, and we have trouble understanding each other”.

When responding to item 3 (they can tell my bad days from my good days) and item 5 (they understand what areas in life I need help with), several of the respondents hesitated and said that they cannot know what the health care workers are thinking and therefore had trouble scoring that item. When replying to item 3, the patients tried to determine if they had noticed any change in the care provided by health care workers, depending on fluctuations in their own wellbeing. When replying to item 5, the respondents explained that they interpreted “in life” as “care needs”.

When responding to item 6 (I’m given enough time to say what I want to say about my health and my care), several of the respondents replied “always” but then added “given the tight time schedule that the health care workers have”. Some respondents concluded that item 9 was the most difficult item to respond to (the staff helps me coordinate my care). Those who did not have a lot of help from the municipal HBPC or those who did not feel that they needed help from the municipal HBPC to coordinate their care found the item irrelevant.

### Surveys in 2022 and 2023

#### Participants

After one reminder, the response rate was 47% (*n* = 1 171) in 2022 and 48% (*n* = 1 429) in 2023. In 2022, 3% (*n* = 33) of the respondents replied to the digital version of the questionnaire, and 8% (*n* = 114) in 2023. When calculating the response rate, patients who passed away during the survey period were excluded, accounting for 1–10% of the population depending on the municipality and year.

Most of the respondents were women (59.5% in 2022, 56.0% in 2023). The median age of the respondents was 84 in 2022 and 83 in 2023, with more than 60% being 80 years or older (2022:64.4%, 2023:60.1%). Less than 10% of the respondents were younger than 65 years (2022:8.9%, 2023:9.4%). Approximately two-thirds of the respondents lived alone (2022:66.7%, 2023:65.4%). Almost three quarters of the respondents received social care services in addition to municipal health care (2022:73.7%, 2023:72.1%). Half of the respondents had received health care and social care services in their home more than three times a day (2022:51.8%, 2023:49.6%). Approximately one quarter of the respondents rated their overall health as poor or fairly poor (2022:24.6%, 2023: 25.4%). More than half of the respondents stated that they suffer somewhat or severely from worry or anxiety (2022:53.8%, 2023:50.2%).

We do not know the exact gender distribution or age range of the population that received the questionnaire. However, we do have data on the gender distribution and age range of all patients who received care in the municipalities included in the study in 2022 and 2023. Based on these figures, men and women appear to have responded to the questionnaire at similar rates. However, the response rate seems to be higher among those aged 80 and over and lower among those aged 18–64.

More information about the sample is presented in Additional file 2.

#### Item and total-score distributions

**Fig. 2:**
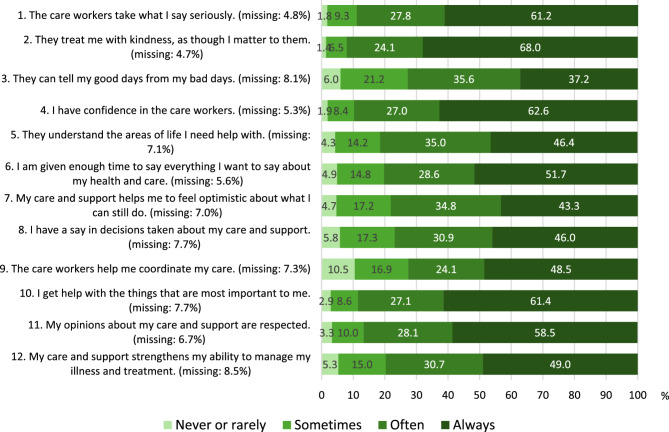
Relative frequency distribution of selected response options and missing responses for each item

The mean PERCCI-S total scores were 39.5 in 2022 and 39.7 in 2023 (based on multiple imputation of missing values). The PERCCI-S total score distributions reveal a ceiling effect, with 18.0% of the respondents scoring the highest possible score (48) and approximately half of the respondents (51.9%) scoring within 7 points of the top of the scale (42–48) when the 2022 and 2023 samples were analysed together. The percentages of item-level missing data ranged from 4.7% (item 2) to 8.5% (item 12). A total of 490 respondents (18.8%) did not score one or more items. A total of 46 respondents (1.5%) omitted one item, 27 respondents (1.1%) omitted two items, and 162 (2.9%) did not score any item but replied to one or more of the other questions in the questionnaire. The relative frequency distributions of selected response options and missing responses for each item in the total dataset are presented in Fig. 2.

#### Factor analysis and item response theory

Exploratory factor analysis of the 2022 data indicated that a dominant first factor (eigenvalue 8.865) accounted for 79% of the variance in the variables. All other factors had eigenvalues < 1. All the variables had significantly high factor loadings. Although the chi-square test had a significant *p* value (χ^2^ = 384.377; df = 54; *p* < 0.001), the RMSEA was 0.073, and the CFI and TLI values were approximately 0.989 and 0.987, respectively, suggesting acceptable model fit.

**Fig. 3:**
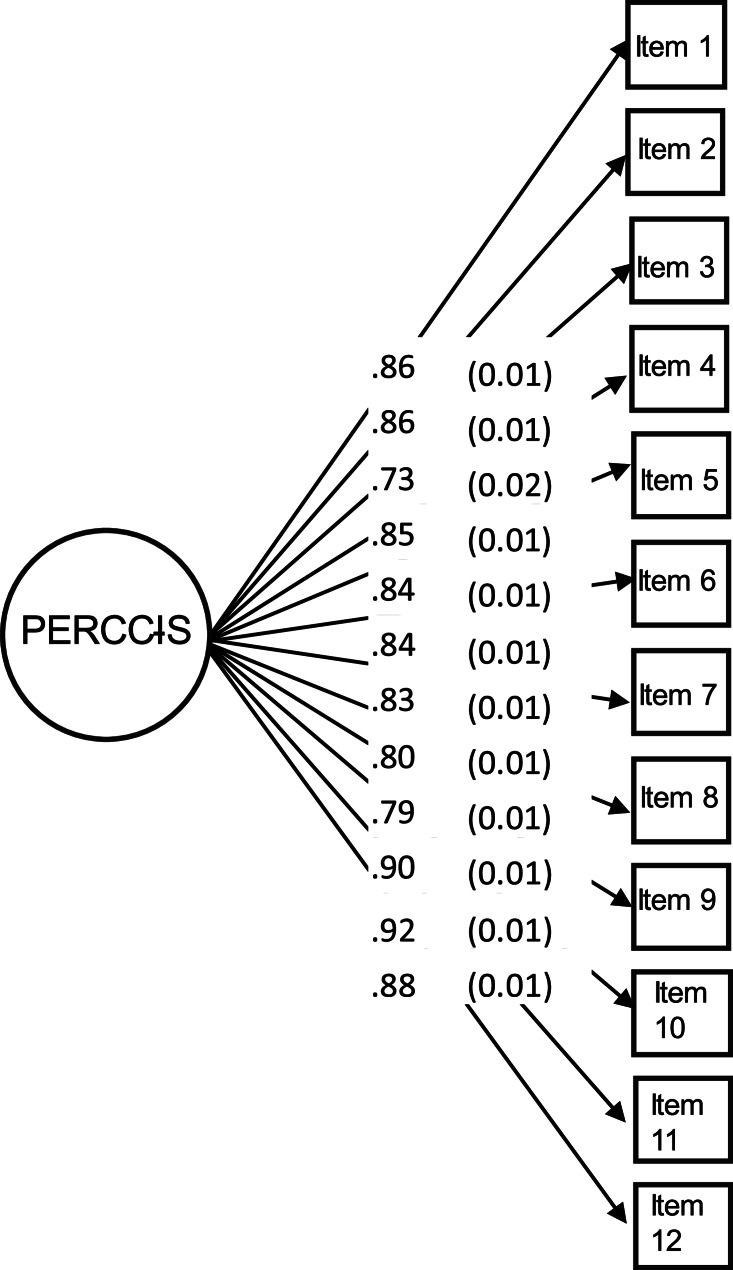
Unidimensional CFA model of PERCCI-S

Confirmatory factor analysis of the unidimensional factor model based on the 2023 data also resulted in acceptable fit (Fig. 3). Although the chi-square coefficient was statistically significant (*p* < 0.001), the fit indices were within an acceptable range (RMSEA = 0.082, CFI = 0.986 and TLI = 0.982). Additionally, scrutiny of the residual correlations (ranging from − 0.071 to 0.073) and modification indices did not reveal areas of substantial misfit.

**Fig. 4:**
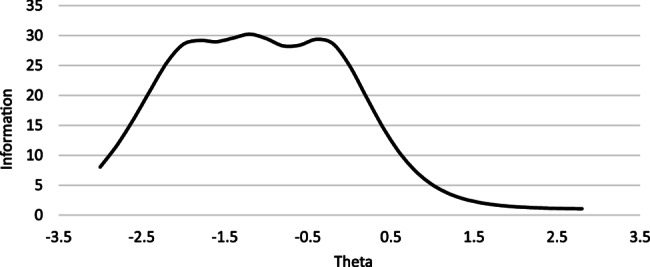
Test information curve for the PERCCI-S item response model

Item Response Theory (IRT) analysis showed that the statistical information provided by the test primarily reflects individuals with lower scores on the latent PERCCI-S scale, meaning the tool is most useful for identifying patients who are dissatisfied with HBPC and do not find it person-centred, but less effective at distinguishing between those who find it moderately or highly person-centred (Fig. 4).

**Fig. 5:**
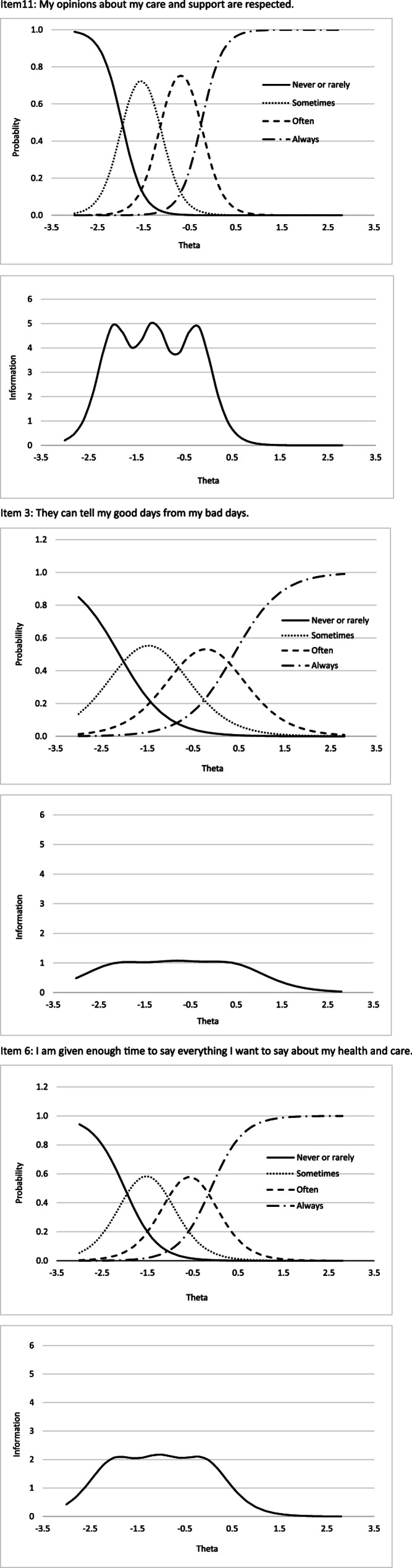
Item response category characteristic curves and information curves for three of the PERCCI-S items

In Fig. 5, we illustrate the information curves for three specific items: one that provides the most information overall, one that provides the least, and one that offers an average level of information. For example, Item 11 (‘My opinions about my care are respected’) provides the highest level of statistical information compared to all other items. However, this information is mostly focused on individuals with lower scores on the PERCCI-S scale. In contrast, Item 3 (‘They can tell my good days from my bad days’) provides the least amount of information overall but offers the most statistical information for individuals with higher scores on the PERCCI-S scale. Finally, Item 6 (‘I am given enough time to say everything I want about my health and treatment’) provides a moderate level of information overall, which is primarily focused on the lower score range of the scale.

#### Reliability

The internal consistency reliability of the PERCCI-S was 0.97 and 0.96 for the 2022 and 2023 surveys, respectively (based on the ordinal alpha of the unidimensional CFAs). The PERCCI-S total score demonstrated excellent test–retest reliability (ICC 0.794). Good test-retest reliability was observed for items 1, 2, 3, 6, 7, 8, 10 and 12. Fair test-retest reliability was observed for items 4, 5, 9 and 11 (Table 1). The same PERCCI-S total score was reported by 29% of the respondents, and 95% of the individual differences between measurements were found to be < 5.22 points. Despite the individual changes between measurements, no significant difference was reported in the mean PERCCI-S total score at the group level between the test and retest measurements, 39.6 ± 8.4 versus 39.7 ± 7.8 (mean ± SD), resulting in a mean difference between the two measurement points of 0.07 points.

**Table 1:** Test-retest reliability of the PERCCI-S using intraclass correlation

Variable	ICC (95% CI)
Total PERCCI-S score	0.79 (0,71-0.86)
Item 1	0.68 (0.56-0,78)
Item 2	0.67 (0.54-0.76)
Item 3	0.60 (0.46-0.72)
Item 4	0.54 (0.38-0.66)
Item 5	0.58 (0.43-0.70)
Item 6	0.63 (0.50-0.74)
Item 7	0.64 (0.50-0.74)
Item 8	0.63 (0.49-0.73)
Item 9	0.53 (0.37-0.66)
Item 10	0.62 (0.47-0.73)
Item 11	0.57 (0.42-0.69)
Item 12	0.62 (0.47-0.73)

Abbreviations: *ICC* intraclass correlation coefficient (based on two-way mixed effects models, absolute agreement, single measure)

#### Convergent and discriminant validity

The correlation of the PERCCI-S total score was greater for overall patient satisfaction with home-based health care (Kendall’s tau-b =.56, *p*<.001), whereas the correlations with perceived overall health, anxiety and frequency of home care were relatively smaller in magnitude (Table 2), thus providing support both convergent and discriminant validity.

**Table 2:** Comparison of mean total PERCCI-S score between groups

Characteristics		N	Mean	Std Dev^1^	Kendall’s tau-b (95% CI)
Control question^2^	Very pleased	1 239	44.1	7.8	-.56 (-.58 to -.54) *p* = <.001
	Quite pleased	854	37.5	7.0	
	Neither pleased nor unpleased	203	30.3	7.4	
	Quite unpleased	62	24.4	8.9	
	Very unpleased	36	19.8	10.7	
	Total	2 417	39,7	8.0	
Perceived overall health	Very good	139	43.1	7.8	-.18 (-.21 to -.15) *p* = <.001
	Fairly good	696	41.4	7.0	
	Neither good or poor	989	39.5	7.4	
	Fairly poor	502	37.1	8.9	
	Very poor	108	37.1	10.7	
	Total	2 434	39.6	8.0	
Anxiety	No problems	1 163	41.3	7.4	-.19 (-.21 to -.21) *p* = <.001
	Yes, some problems	1 037	38.7	8.0	
	Yes, severe problems	225	35.8	9.2	
	Total	2 425	39.7	8.0	
Frequency of care	3 or more times a day	1 221	39.1	8.0	.09 (.06 -.12) *p* = <.001
	1–2 times a day	571	39.9	7.9	
	More than once a week	222	41.3	7.3	
	Once a week	142	42.5	6.4	
	Less than once a week	177	40.1	8.3	
	Total	2 333	39.7	8.0	

^1^Average standard deviation across imputations

^2^The control question was as follows: How pleased are you in general with the health care you receive in your home?

Note: Results based on the combined dataset (2022 and 2023 data) using multiple imputations to handle missing data for computing the PERCCI-S total score

#### Methods for handling missing data

The comparison between different methods of handling missing data shows that replacing missing data with the average mean or 10 imputations produces almost the same mean total PERCCI-S score on a municipal level. Using listwise deletion to handle missing data resulted in higher total PERCCI-S scores for all municipalities except one than using the average mean or 10 imputations.

## Discussion

Building on the 12-item version of PERCCI as a foundation, this study describes the adaptation, further development, and validation of the PERCCI-S, a tool designed to measure PCC in HBPC within the Swedish municipal health care system.

The novelty of PERCCI-S lies not in the development of a wholly new conceptual framework for person-centeredness but in its targeted application to home-care services - a domain that has historically lacked validated instruments. PERCCI-S was developed with input from patients receiving home-based care, addressing the unique dynamics of delivering care in patients’ homes where the person often meets several different health care workers and receives frequent interventions over a longer period. PERCCI-S fills this gap by operationalizing aspects of care delivery that are critical for HBPC, such as respect for the patient’s autonomy in their home environment and the integration of social welfare considerations into care planning. Previous studies on PCC show that patients seem to prioritize informal aspects of their relationship with healthcare providers, such as fostering a human connection, over more formal elements like goal setting and documentation [54].By emphasizing relational and interpersonal aspects of care, PERCCI-S aligns closely with patient priorities in HBPC, where trust and respect are paramount.

Results from the focus groups with professionals in phase one were essential to gain insight into how the 12-item PERCCI should be revised to work in a Swedish context. For example, while embracing the importance of a trustful relationship between patient and care workers as emphasized in the literature as central dimensions to PCC [9, 10], the participants underlined that the relationship must be professional, which resulted in alterations to item 4. The participants also pointed out that coordinating care is a core responsibility for municipal HBPC in Sweden, resulting in a new focus for item 9. The cognitive interviews with patients and the expert panel results demonstrated good content validity overall. However, item 9 (The care workers help me coordinate my care) was identified in the patient interviews as challenging to answer for those who did not feel they needed assistance with care coordination. Additionally, the interviews highlighted that patients consider continuity of care a key element of PCC, along with healthcare workers being well-informed about the patient’s situation and care—elements not currently addressed in the PERCCI-S. During the expert panel discussion, some participants suggested revising items 8 and 11 to present the patient as an equal partner working with healthcare professionals to achieve care goals. This change would better align with the contemporary Swedish model of PCC, which emphasizes the patient’s role as an active collaborator in the care process [10, 53].

The PERCCI-S demonstrated a moderate amount of missing data, and the response rates were good, especially considering that the instrument was distributed through a postal questionnaire, that the mean age of the respondents was over 80 years, and that patients with cognitive disabilities were included. The response rate in this study was much higher (2022:47%; 2023: 48%) than the response rate in the study of the original PERCCI [26] (29%) and comparable to or higher than the response rates of other patient questionnaires in Sweden [55, 56]. This finding indicates that PERCCI-S and the items included were easy to understand and could be perceived as meaningful, a conclusion also supported by the result of the cognitive interviews with patients.

While it is quite typical for PREMs and PCC instruments to yield results that are indicative of positive experiences and to have notable ceiling effects [57, 58], these are nonetheless significant concerns that can hinder efforts to improve care [40]. The presence of such effects suggests that extreme items may be absent at the upper end of the scale, possibly indicating restricted content validity. Consequently, patients scoring at the extremes cannot be differentiated from one another, leading to reduced reliability. Additionally, responsiveness is diminished, as changes in these patients cannot be effectively measured. The PERCCI-S total score distributions reveal a ceiling effect, which implies that the tool is predominantly useful for identifying patients who are displeased with HBPC and who do not find it person-centred. The PERCCI-S is, however, less able to identify patients who find HBPC to be very person-centred in comparison to those who find it moderately person-centred. This means that the PERCCI-S is potentially a particularly useful tool for quality improvement in municipalities and departments where patients are unsatisfied with the quality of care but is less suitable for evaluating whether improvement work leads to very, as opposed to moderate, person-centred care.

EFA and CFA indicate a dominant single dimension. Test‒retest reliability revealed good temporal stability. Convergent validity was supported for the PERCCI-S since the total score correlated moderately with the single question on overall patient satisfaction. The PERCCI-S showed good discriminant validity in distinguishing patients on the basis of perceived overall health, problems with worry or anxiety and frequency of homecare.

The PERCCI-S showed good internal consistency reliability, with ordinal alphas of 0.97 and 0.96 for the 2022 and 2023 samples, respectively. High alpha values can indicate problems with redundancy, and the literature commonly warns about alpha values above 0.9 or 0.95. However, some authors state that alpha values above 0.9 are desirable for instruments that are used to make individual decisions [59]. Despite high internal consistency for the total PERCCI-S score, we found no signs of redundancy in discussions with the expert panel or in the cognitive interviews with patients.

In general, the listwise deletion method of handling missing data results in excessively high PERCCI-S scores and should not be used. Theoretically, multiple imputation would produce the most trustworthy results. However, replacing missing data with the average mean for those replying to half or more of the items (a.k.a. mean imputation) produced almost the same mean total PERCCI-S score in our study. Thus, if multiple imputation is not feasible, municipalities can consider mean imputation for the purpose of calculating point estimates.

### Strengths and limitations

A strength of this study is the large sample collected over two years and the application of several methods to collect and analyse qualitative and quantitative data to explain, extend and validate the questionnaire’s measurement properties. Another strength is that we used statistical analyses appropriate for ordinal data [59] and that we used appropriate missing data methods to include questionnaires in our analyses where respondents omitted replying to one or more items.

We acknowledge that certain items in PERCCI-S may have broader applicability than municipal HBCP and could potentially be adjusted for other care settings. This is a strength rather than a limitation, as it allows for adaptability while maintaining focus on municipal primary care, regardless of whether it is given in the patient’s home or in an institutional setting.

Throughout the process, we made efforts to involve patients. The absence of patient involvement in the revision of PERCCI during phase one can be seen as a limitation. The main limitation of our study is however that it did not allow for a drop-out analysis for aspects other than age and sex. For example, we do not know if patients with cognitive disabilities or patients who do not have Swedish as their mother tongue replied to the questionnaire to the same extent. Another limitation is that part of the sample includes patients who participated in both surveys. The results for the two years may be more similar than if we had used independent samples. However, because the surveys were conducted one year apart, we believe the impact of this overlap is minimal.

### Recommendations for further research

Measurement validation is an ongoing process that requires the accumulation of different forms of evidence to support the use of a measure for its intended purposes [60–66]. From this viewpoint, ongoing generation of new validity evidence is necessary to support the adequacy and appropriateness of the use of PREMs and PCC instruments in languages and cultural contexts other than where they were originally developed and tested. We recommend that further research focus on whether different groups of people (e.g., younger vs older respondents, respondents from different cultures) interpret and respond consistently to the PERCCI-S items via differential item function analyses. Future research should also focus on how useful stakeholders find the PERCCI-S as a basis for quality improvement and decision making.

Although the PERCCI-S has good psychometric properties, evidence from this study suggests that some future alterations may help to further improve the measurement. For example, it may be beneficial to revise item 9 (the care workers help me coordinate my care) by more explicitly focusing on continuity of care. It may also be advantageous to change the wording of items 8 (My care and support helps me to feel optimistic about what I can still do) and 11 (My opinions about my care and support are respected) to better align with the contemporary model of PCC, viewing the patient as an active collaborator [53]. Furthermore, researchers have suggested that adding more response options to the present 4-grade Likert scale or changing the response scale may help reduce ceiling effects [67–70]. We also want to point out that the measure has undergone modifications from the original instrument developed in Great Britain to ensure contextual validity and to make some of the items easier for patients to understand. The measure is notably different from the original measure, making it unsuitable for comparing results between Sweden and Great Britain.

## Conclusion

In general, the PERCCI-S demonstrates good psychometric properties, indicating that it is potentially a relevant measure of patients' experiences of PCC in HBPC. For the time being, we consider the PERCCI-S merits as a measure of quality and benchmarking of PCC in the Swedish municipal HBPC. It seems to be a particularly useful tool for identifying patients who are less satisfied with the quality of care.

## Supplementary Information

Supplementary Material 1: Different versions of the PERCCI and the PERCCI-S

Supplementary Material 2: Respondent characteristics

## Data Availability

The datasets generated and/or analysed during the current study are not publicly available owing to confidentiality, but excerpts are available from the corresponding author upon reasonable request.

## Abbreviations

PCC: Person-Centred Care
GPCC: The Gothenburg University Centre for Person-Centred Care
HBPC: Home-based primary care
CVI: Content validity index
EFA: Exploratory factor analysis
CFA: Confirmatory factor analysis
IRT: Item response theory
ICC: Intraclass correlation coefficient
