# Supplementary material for: Adaptation, further development and evaluation of the measurement properties of the person-centred community care inventory (PERCCI-S) for use in the Swedish municipal health care system
Source: BMC Health Serv Res. 2025 Jul 30;25:1004. doi: 10.1186/s12913-025-13178-7 (PMC12312282; doi:10.1186/s12913-025-13178-7)
Supplement: Supplementary file 1 — Supplementary Material 1: Different versions of the PERCCI and the PERCCI-S [file 12913_2025_13178_MOESM1_ESM.docx]

# Additional file 1

**Different versions of PERCCI and the PERCCI-S**

| Original version presented in Wilberforce et al. (2018) | 12-item version published on PERCCI.org | The PERCCI-S |
| --- | --- | --- |
| 1. They show an interest in me as a person |  |  |
| 2. They know me well enough to recognize when I’m feeling down |  |  |
|  | 1. My care workers take what I have to say seriously | 1. The care workers take what I say seriously. |
|  | 2. They treat me with kindness, as though I matter to them | 2. They treat me with kindness, as though I matter to them. |
| 3. They can tell my good days from my bad days | 3. They can tell my good days from my bad days | 3. They can tell my good days from my bad days. |
| 4. I have developed a close connection with them | 4. I feel I have developed a close connection with my care workers | 4. I have confidence in the care workers. |
| 5. They are genuinely caring, not just going through the motions |  |  |
| 6. They really understand me |  |  |
| 7. They understand the areas of my life that I need help with | 5. They understand the areas of my life that I need help with | 5. They understand the areas of life I need help with. |
| 8. I am given enough time to say everything that I want to say | 6. I am given enough time to say everything that I want to say | 6. I am given enough time to say everything I want to say about my health and care. |
| 9. They speak to me in a friendly and respectful manner |  |  |
| 10. I have a say in decisions taken about my care and support | 7. I have a say in decisions taken about my care and support. | 7. I have a say in decisions taken about my care and support. |
| 15. My care and support helps me to feel optimistic about what I can still do | 8. My care and support helps me to feel optimistic about what I can still do | 8. My care and support helps me to feel optimistic about what I can still do. |
| 11. I am given opportunity to join groups where I can meet other people | 9. I am helped to keep in touch with my local community | 9. The care workers help me coordinate my care. |
| 12. I get help with things that are most important to me | 10. I get help with the things that are most important to me | 10. I get help with the things that are most important to me. |
| 13. My opinions about my care and support are respected | 11. My opinions about my care and support are respected | 11. My opinions about my care and support are respected. |
| 14. They are interested in my views about my care and support |  |  |
| 16. My care and support helps me to build confidence | 12. My care and support helps me to build confidence. | 12. My care and support strengthens my ability to manage my illness and treatment. |
| 17. I feel that I must do as I’m told |  |  |
| 18. I see too many different staff |  |  |
| 19. Services are too focused on the paperwork, rather than the care |  |  |
