# Supplementary material for: Adaptation, further development and evaluation of the measurement properties of the person-centred community care inventory (PERCCI-S) for use in the Swedish municipal health care system
Source: BMC Health Serv Res. 2025 Jul 30;25:1004. doi: 10.1186/s12913-025-13178-7 (PMC12312282; doi:10.1186/s12913-025-13178-7)
Supplement: Supplementary file 2 — Supplementary Material 2: Respondent characteristics [file 12913_2025_13178_MOESM2_ESM.docx]

**Additional file 2 Respondent characteristics**

| **Respondent characteristics** | **Study 2022 (n=1 171)** | | **Study 2023 (n=1 429)** | |
| --- | --- | --- | --- | --- |
|  | **Respondents (n)** | **Respondents (%)** | **Respondents (n)** | **Respondents (%)** |
| **Gender** | missing = 43 | | missing = 55 | |
| Female | 671 | 59.5 | 770 | 56.0 |
| Male | 457 | 40.5 | 602 | 43.8 |
| Other | - | - | 2 | 0.1 |
| **Age** | missing = 27, range = 2 6 - 105, mean = 81,  median = 84 | | missing = 95, range = 25 – 106, mean = 80,  median = 83 | |
| Under 65 | 102 | 8.9 | 125 | 9.4 |
| 65-69 | 65 | 5.7 | 89 | 6.2 |
| 70-79 | 241 | 21.1 | 317 | 23.8 |
| 80-89 | 462 | 40.4 | 511 | 38.3 |
| 90 or over | 274 | 24.0 | 292 | 21.9 |
| **How do you rate your overall health?** | missing = 49 | | missing = 57 | |
| Very good | 68 | 6.1 | 76 | 5.5 |
| Fairly good | 330 | 29.4 | 379 | 27.6 |
| Fair | 448 | 39.9 | 568 | 41.4 |
| Fairly poor | 223 | 19.9 | 290 | 21.1 |
| Poor | 53 | 4.7 | 59 | 4.3 |
| **Do you suffer from anxiety?** | missing = 53 | | missing = 65 | |
| No | 517 | 46.2 | 679 | 47.8 |
| Yes, somewhat | 493 | 44.1 | 560 | 41.1 |
| Yes, severely | 108 | 9.7 | 125 | 9.1 |
| **Do you live with another adult?** | missing = 46 | | missing = 55 | |
| Yes | 375 | 33.3 | 475 | 34.6 |
| No | 750 | 66.7 | 899 | 65.4 |
| **Do you also receive social services in your home?** | missing = 76 | | missing = 58 | |
| Yes | 806 | 73.6 | 988 | 72.1 |
| No | 257 | 23.5 | 328 | 23.9 |
| I don’t know | 32 | 2.9 | 55 | 4.0 |
| **For how long have you received health care in your home?** |  | | missing = 55 | |
| Less than 1 month |  |  | 40 | 2.9 |
| 1 to 6 months |  |  | 216 | 15.7 |
| 6 months to 1 year |  |  | 257 | 18.7 |
| More than 1 year |  |  | 809 | 58.9 |
| …I don’t know |  |  | 52 | 3.8 |
| **How often do your receive health care and social services in your home?** | missing = 79 | | missing = 67 | |
| 3 or more times per day | 566 | 51.8 | 675 | 49.6 |
| 1-2 times a day | 258 | 23.6 | 325 | 23.9 |
| More than once a week | 82 | 7.5 | 147 | 10.8 |
| Once a week | 72 | 6.6 | 73 | 5.4 |
| Less often than once a week | 88 | 8.1 | 95 | 7.0 |
| …I don’t know | 26 | 2.4 | 47 | 3.5 |
| **How pleased are you in general with the health care you receive in your home?** | missing = 79 | | missing = 70 | |
| Very pleased | 548 | 49.1 | 707 | 53.0 |
| Quite pleased | 407 | 36.4 | 461 | 33.9 |
| Neither pleased nor unpleased | 100 | 9.0 | 112 | 8.2 |
| Quite unpleased | 31 | 2.8 | 35 | 2.6 |
| Very unpleased | 21 | 1.9 | 14 | 1.0 |
| …I don’t know | 10 | 0.9 | 17 | 1.3 |
| **Did someone assist you in replying to the questionnaire?** | missing = 78 | | missing = 71 | |
| No, I replied on my own. | 486 | 44.5 | 606 | 44.6 |
| Yes, someone assisted. | 607 | 55.5 | 752 | 55.4 |
